# Molecular characterization of *rpoB* gene mutations in isolates from tuberculosis patients in Cubal, Republic of Angola

**DOI:** 10.1186/s12879-021-06763-8

**Published:** 2021-10-12

**Authors:** Ariadna Rando-Segura, María Luisa Aznar, María Milagros Moreno, Mateu Espasa Soley, Elena Sulleiro Igual, Cristina Bocanegra Garcia, Eva Gil Olivas, Arlete Nindia Eugénio, Carlos Escartin Huesca, Adriano Zacarias, Josep Vegue Collado, Domingos Katimba, Maria Carmen Vivas Cano, Estevao Gabriel, Maria Teresa López García, Tomas Pumarola Suñe, Israel Molina Romero, María Teresa Tórtola Fernández

**Affiliations:** 1 Microbiology Department, Vall d’Hebron University Hospital, PROSICS Barcelona, Universitat Autònoma de Barcelona, Passeig Vall d’Hebron 119 – 129, 08035 Barcelona, Spain; 2 Infectious Disease Department, Vall d’Hebron University Hospital, PROSICS Barcelona, Universitat Autònoma de Barcelona, Barcelona, Spain; 3 Hospital Nossa Senhora da Paz, Cubal, Angola

**Keywords:** Angola, Cubal, GenoType MTBDRplus VER2.0, Resistance, Rifampicin, *rpoB* mutations

## Abstract

**Background:**

The importance of *Mycobacterium tuberculosis* strains with disputed *rpoB* mutations remains to be defined. This study aimed to assess the frequency and types of *rpoB* mutations in *M. tuberculosis* isolates from Cubal, Angola, a country with a high incidence of tuberculosis.

**Methods:**

All isolates included (n = 308) were analyzed using phenotypic drug susceptibility testing and GenoType MTBDR*plus* assay. DNA sequencing of the *rpoB* gene and determination of rifampicin MIC by macrodilution method were additionally performed on isolates yielding discordant results (n = 12) and those in which the mutation detected was not characterized (n = 8).

**Results:**

In total, 85.1% (74/87) of rifampicin-resistant strains had undisputed rpoB mutations -S450L (49), D435V (15), H445D (3), H445Y (2), Q432ins (1), L449M plus S450F (1), S450F (1), S450W (1) and S450Y (1)-; 10.3% (9/87) had disputed rpoB mutations—L430P plus S493L (1), N437del (1), H445L (3), D435Y (2), L452P (2)-, 2.3% (2.3%) showed no *rpoB* mutations and 2.3% (2/87) showed heteroresistance—D435Y plus L452P and L430P plus S493L-.

**Conclusion:**

Disputed rpoB mutations were common, occurring in 10.3% of rifampicin resistant isolates. Current phenotyping techniques may be unable to detect this resistance pattern. To increase their sensitivity, a lower concentration of RIF could be used in these tests or alternatively, *rpoB* mutations could be screened and characterized in all *M. tuberculosis* strains.

## Background

The emergence of resistance to anti-tuberculosis drugs, and particularly multidrug-resistant (MDR) tuberculosis (TB)—defined as resistance to both isoniazid (INH) and rifampicin (INH)—is a major public health problem in many countries and an important obstacle to effective global TB control [1]. Effective management of TB relies on a prompt diagnosis, rapid detection of drug resistance, and fast initiation of an effective treatment regimen [2]. Resistance to RIF is the most important indicator of MDR-TB [3]. RIF resistance is mainly associated with single point mutations in a small 81 base pair (bp) hotspot region of the *rpoB* gene, referred to as the RIF-resistance-determining region (RRDR), which can easily be amplified by PCR [4]. Thus, several molecular genetic tests to detect RRDR mutations have been developed and evaluated for their ability to detect resistance in clinical isolates (e.g. GenoType MTBDR*plus*, Hain Lifescience; Xpert MTB/RIF and Xpert Ultra, Cepheid; Truenat MTB-RIF Dx, Molbio Diagnostics) [2].

Broad use of molecular assays invariably find isolates harboring *rpoB* mutations that did not test resistant by culture-based phenotypic drug susceptibility testing (DST) methods [5–10]. Culture-based DST is considered the gold standard for this purpose. However, a study carried out by Supranational Reference Laboratories (SRL) showed that mutations in *rpoB* associated with low-level RIF resistance are easily missed by culture-based phenotypic DST methods, in particular automated broth-based Bactec Systems [2, 11]. What remains to be resolved is the importance of discordant strains with disputed *rpoB* mutations in terms of their relative frequency and impact on the outcome of RIF-based antituberculosis therapy [12]. In general they are considered rare, and their minimum inhibitory concentration (MIC) is usually below the critical concentration [12]. For this reason, they are often considered RIFusceptible, although there are reports of adverse treatment outcomes [12, 13].

The aims of our study were to determine the prevalence of *rpoB* mutations (undisputed, disputed, or silent), the association between *rpoB* mutations and the RIF MIC and treatment outcomes in cases with disputed and undisputed mutations in Cubal, Republic of Angola, a country with a high TB incidence.

## Methods

### Study design and data collection

This study included 308 *Mycobacterium tuberculosis* complex isolates, 225 from new cases and 83 from retreatment cases. Samples were collected from patients with TB attended in Hospital Nossa Senhora da Paz (Cubal, Angola) and sent to the Mycobacteriology Unit (WHO Supranational TB Reference Laboratory in Spain) of Vall d’Hebron University Hospital for culture and DST. Details of the inclusion and exclusion criteria and sample shipping have been previously described by our research team [14]. DNA sequencing of the *rpoB* gene and MIC determination by the macrodilution method were performed on isolates that showed RIF resistance by culture-based DST, but were identified as susceptible by GenoType MTBDRplus Hain Lifescience or did not give hybridization results with the mutation-specific probes, and on those that showed RIF susceptibility by culture-based DST but were identified as resistant by molecular methods.

### rpoB sequencing

DNA extraction was carried using organic-solvent (chloroform-isoamyl alcohol). A 1428-bp region from codon 135 to 610 was sequenced, as this region has been described as containing mutation conferring RIF resistance. Amplification of the *rpoB* gene was carried out with a conventional PCR assay using the Expand High Fidelity PCR kit (ROCHE, Basel, Switzerland), primers and PCR protocols (Table 1). PCR products were purified using Exo-SAP-IT (Affymetrix, Cleveland, Ohio, USA) and sequenced using the ABI Prism BigDye Terminator cycle sequencing kit v3.1 on the ABI PRISM 3130XL sequencer (Applied Biosystems, Foster City, California, USA). The same primers used for amplification were also used for sequencing. Nucleotide sequences were assembled and edited using MEGA X: Molecular Evolutionary Genetics Analysis across computing platforms. Consensus sequences from each sample were compared to the MTB H37Rv reference sequence (GenBank: AL123456). The numbering system used in the results has been described previously for MTB H37Rv [15].

**Table 1:** Primers and PCR amplification and sequencing protocols of partial *rpoB* gene sequence

Gene	Fragment	Primers	PCR condition	Position (aa)
*rpoB*	1	*rpoB*-1F: CAACAACAACACCGGTGAGA *rpoB*-1R: CTGGTTTTGGATCAGCTCGC	94 °C × 5′ 45c: (94 °C × 45″–66 °C c 1′–72 °C × 45″) 72 °C × 10′	135–382
	2	*rpoB*-2F: GTACGGTCGGCGAGCTGA *rpoB*-2R: CCTGGCGCTGCATGTTTG	94 °C × 5′ 45c: (94 °C × 45″–66 °C c 1′–72 °C × 45″) 72 °C × 10′	376–610

*aa* aminoacid

### Rifampicin minimum inhibitory concentration

Pure RIF powder was dissolved in dimethyl sulfoxide according to the manufacturer’s (Sigma-Aldrich, St. Louis, MO, USA) instructions at a concentration of 100 mg/ml with subsequent dilutions in sterile distilled water.

Bacterial suspensions were prepared in Middlebrook 7H9 and adjusted to an opacity equal to McFarland standard 0.5. The bacterial suspension was used for the primary culture required for further testing in the Bactec MGIT 960 system (Becton Dickinson Diagnostic Systems, Sparks, MD). Testing for RIF MICs on MGIT 960 systems was done using the following concentrations: 0.125, 0.25, 0.5, 1.0, 2.0 and 4.0 μg/ml.

### Data analysis

Statistical analyses were performed using the Stata 12 software (StataCorp. 2011. Stata Statistical Software: Release 12. College Station, TX: StataCorp LP). *Missed resistance* corresponds to isolates testing false-susceptible divided by the *corrected phenotype*, comprising the sum of phenotypically resistant isolates and false-susceptible isolates.

## Results

### Phenotypic RIF susceptible cases

Overall, 227 of the total 308 isolates were found to be RIF-susceptible by culture-based DST. Eight isolates showing phenotypic susceptibility to RIF were identified as resistant by MTBDR*plus*. The mutations detected by sequencing of these discordant isolates were *rpoB* L430P (1), D435Y (2), H445L (2), H445N (1) and L452P (2); and the distribution of MICs ranged from 0.125 to 1.0 μg/ml (one isolated, carrier of the L452P mutation, failed to grow) (Table 2).

**Table 2:** *rpoB* mutations found among culture-positive phenotypic RIF-susceptible cases

Frequency		Culture-based DST (MGIT 960)		MIC (MGIT 960)	GenoType MTBDR*plus*			Sequencing
		INH	RIF	RIF	*rpoB*	*katG*	*inhA*	*rpoB*
176	C1	S	S	Not done	wt pattern	wt pattern	wt pattern	Not done
10	C1	R	S	Not done	wt pattern	∆wt, S315T1	wt pattern	Not done
1	C1	R	S	Not done	wt pattern	∆wt	wt pattern	Not done
2	C1	R	S	Not done	wt pattern	wt pattern	∆wt1, C15T	Not done
13	C1	R	S	Not done	wt pattern	wt pattern	wt pattern	Not done
1	C1	R	S	0.125	∆wt2	∆wt, S315T1	wt pattern	L430P (+)
1	C1	R	S	0.125	∆wt7	wt pattern	wt pattern	H445N (+)
1	C1	R	S	0.5	∆wt7	∆wt, S315T1	wt pattern	H445L (+++)
15	C2	S	S	Not done	wt pattern	wt pattern	wt pattern	Not done
1	C2	R	S	Not done	wt pattern	∆wt, S315T1	wt pattern	Not done
1	C2	R	S	Not done	wt pattern	wt pattern	wt pattern	Not done
2	C2	R	S	0.5	∆wt3,4	∆wt, S315T1	wt pattern	D435Y (++)
1	C2	R	S	1.0	∆wt7	∆wt	wt pattern	H445L (+++)
1	C2	R	S	0.5	∆wt8	∆wt, S315T1	wt pattern	L452P (++)
1	C2	R	S	No viable	∆wt8	∆wt	wt pattern	L452P (++)

*DST* drug susceptibility testing; *MIC* minimum inhibitory concentration; *C1* new cases; *C2* previously treated cases

WT1 (424–428), WT2 (429–432), WT3 (429–436), WT4 (435–438), WT5 (437–441), WT6 (441–444), WT7 (445–448), WT8 (449–452)

(+++) high confidence for association with resistance, (++) moderate confidence for association with resistance, (+) minimal confidence for association with resistance

### Phenotypic RIF resistant cases

Overall, 81 isolates were found to be RIF-resistant by culture-based DST, and 77 were identified as resistant by MTBDR*plus*. Of the latter, 69 gave positive hybridization results with the mutation-specific probes S450L (49), D435V (15), H445D (3) and H445Y (2). The remaining 8 isolates were identified as resistant by the absence of the wild-type probes [14]. Mutations detected by sequencing were *rpoB* L430P plus S493L (1), Q432ins (1), N437del (1), H445L (1), L449M plus S450F (1), S450F (1), S450W (1), and S450Y (1), and the distribution of MICs among these isolates ranged from 1.0 to > 4.0 μg/ml. Mutations at codon 450 and insertions tend to cause high or moderate levels of resistance (MIC ≥ 4.0 μg/ml), whereas H445L, double mutants and, N437del tend to cause low resistance (MIC = 1.0 to 2.0 μg/ml). Thus, one twofold MIC dilution, which is considered acceptable, can change the categorical result between resistant and susceptible. The remaining 4 isolates were identified as susceptible by MTBDR*plus*. Sequencing of these isolates showed that 2 of them had no *rpoB* mutations, and mutations detected in the other 2 isolates were *rpoB* D435Y plus L452P and L430P plus S493L. These mutations should have been detected by an absence of the wild-type hybridization signal; non-detection suggests heteroresistance (Table 3).

**Table 3:** *rpoB* mutations found among culture-positive phenotypic RIF-resistant cases

Freq		Culture-based DST (MGIT 960)		CMI (MGIT 960)	GenoType MTBDR*plus*			Sequencing
		INH	RIF	RIF	*RpoB*	*KatG*	*inhA*	*RpoB*
1	C1	R	R	Not done	Δwt2,3,4, D435V	∆wt, S315T1	wt pattern	Not done
2	C1	R	R	Not done	Δwt3,4, D435V	∆wt, S315T1	wt pattern	Not done
1	C1	R	R	Not done	Δwt3,4, D435V	wt pattern	wt pattern	Not done
2	C1	S	R	Not done	Δwt7, H445Y	wt pattern	wt pattern	Not done
1	C1	R	R	> 4. 0 μg/ml	Δwt8	wt pattern	wt pattern	L449M + S450F (+++)
8	C1	R	R	Not done	Δwt8, S450L	∆wt, S315T1	wt pattern	Not done
2	C1	R	R	Not done	Δwt8, S450L	∆wt	wt pattern	Not done
2	C1	R	R	Not done	Δwt8, S450L	wt pattern	wt pattern	Not done
1	C1	R	R	> 4. 0 μg/ml	wt pattern	wt pattern	wt pattern	NO mutation
1	C2	R	R	4.0 μg/ml	wt pattern	∆wt, S315T1	wt pattern	L430P* + S493L*
1	C2	R	R	2.0 μg/ml	Δwt2	∆wt, S315T1	wt pattern	L430P + S493L
1	C2	R	R	0.5 μg/ml	wt pattern	∆wt, S315T1	wt pattern	D435Y* + L452P*
1	C2	R	R	4.0 μg/ml	Δwt2	wt pattern	wt pattern	Q432ins
8	C2	R	R	Not done	Δwt2,3,4, D435V	∆wt, S315T1	wt pattern	*Not done*
2	C2	R	R	Not done	Δwt3,4, D435V	∆wt, S315T1	wt pattern	Not done
1	C2	R	R	Not done	Δwt3,4, D435V	wt pattern	wt pattern	Not done
1	C2	R	R	1. 0 μg/ml	Δwt4,5	wt pattern	wt pattern	N437del (+++)
1	C2	R	R	Not done	Δwt7, H445D	∆wt, S315T1	wt pattern	Not done
1	C2	R	R	Not done	Δwt7,8, H445D	∆wt, S315T1	wt pattern	Not done
1	C2	R	R	Not done	Δwt7, H445D	wt pattern	wt pattern	Not done
1	C2	R	R	2. 0 μg/ml	Δwt7	∆wt	wt pattern	H445L (+++)
1	C2	R	R	> 4. 0 μg/ml	Δwt8	∆wt, S315T1	wt pattern	S450Y (+++)
1	C2	R	R	> 4. 0 μg/ml	Δwt8	wt pattern	∆wt1, C15T	S450F (+++)
1	C2	R	R	> 4. 0 μg/ml	Δwt8	wt pattern	wt pattern	S450W (+++)
22	C2	R	R	Not done	Δwt8, S450L	∆wt, S315T1	wt pattern	*Not done*
2	C2	R	R	Not done	Δwt8, S450L	∆wt	wt pattern	Not done
1	C2	R	R	Not done	Δwt8, S450L	wt pattern	∆wt1, C15T	Not done
1	C2	R	R	Not done	Δwt8, S450L	wt pattern	∆wt1	Not done
1	C2	R	R	Not done	Δwt8, S450L	wt pattern	C15T	Not done
8	C2	R	R	Not done	Δwt8, S450L	wt pattern	wt pattern	Not done
2	C2	S	R	Not done	Δwt8, S450L	wt pattern	wt pattern	Not done
1	C2	R	R	< 0.13 μg/ml	wt pattern	wt pattern	wt pattern	NO mutation

*DST* drug susceptibility testing; *MIC* minimum inhibitory concentration; *C1* new cases; *C2* previously treated cases

WT1 (424–428), WT2 (429–432), WT3 (429–436), WT4 (435–438), WT5 (437–441), WT6 (441–444), WT7 (445–448), WT8(449–452)

*Heteroresistance; (+++) high confidence for association with resistance, (++) moderate confidence for association with resistance, (+) minimal confidence for association with resistance

### Strains with disputable mutations

Disputed mutations were defined as RRDR mutations in isolates having a RIF MIC of 0.125–2.0 μg/ml. Eleven isolates were considered to have disputed mutations (even though one isolate failed to grow): 8 were phenotypic RIFsusceptible cases and 3 were phenotypic RIF-resistant cases. All of them were also resistant to INH. Three of these isolates (3/225, 1.3%) were from new cases and patients received Category 1 treatment regimens, whereas the other 8 (8/83, 9.6%) were from retreatment cases and patients received second-line TB medications. Among the 3 new cases that received Category 1 treatment regimens, at the end of treatment, 1 (33.3%) patient had treatment success, 1 (33.3%) was lost to follow-up, and 1 (33.3%) died. Among the 8 retreatment cases that received second-line TB medications, at the end of treatment, 4 (50.0%) patients had treatment success, 1 (12.5%) was lost to follow-up, and 3 (37.5%) died.

## Discussion

Commercial molecular techniques are very accurate for detecting RIF resistance, but discordance between molecular and phenotypic methods invariably occurs. The reasons for discordant results range from disputed mutations with an increased MIC below the critical concentration in some DST culture systems, mutations outside the RRDR, silent mutations and heteroresistance [16, 22].

Mutations in *rpoB* can confer different levels of RIF resistance [10]. The position of the mutation and the amino acid change it causes determine the resistance level. Some mutations are associated with high levels of resistance (e.g. S450L, H445Y and H445D), whereas others tend to cause moderate or low resistance (D435V, D435Y, H445L and L452P respectively) [17]. We observed 11 strains with disputed mutations (carriers of mutations in RRDR and MIC ≤ 2.0 μg/ml), all of them also resistant to INH. The majority (8/11, 72.7%) of patients with strains showing disputed mutations had a previous history of TB; hence, accumulation of resistance due to prior exposure to TB treatment may have contributed to their development.

Strains with disputed mutations showed MICs bordering the critical concentration of 1.0 μg/ml. These strains present two challenges for cultured based methods. The first concerns the reproducibility of the results, because even a small variation in the MIC (one twofold MIC dilution), which is considered acceptable, can change the categorical result of resistant or susceptible. The second problem is due to the overlap of MICs between discordant and wild-type isolates [10]. In our study, the distribution of MICs in discordant isolates ranged from 0.125 to 1.0 μg/ml, but only some of these discordant mutations have been clearly associated with resistance. The H445L mutation has high confidence for association with resistance, mutations D435Y and L452P have moderate confidence, and L430P and H445N have minimal confidence for association with resistance, and therefore, inconclusive evidence that the mutation confers or is strongly associated with drug resistance [18]. These associations correlated well with the MICs of these isolates. The H445L, D435Y, and L452P mutants tend to cause low level resistance (MIC 0.5–1.0 μg/ml), whereas isolates with the H445N and L430P had MICs under the epidemiological cutoff (ECOFF) of 0.25–0.5 μg/ml (MIC 0.125 μg/ml). Isolates with mutations having high or moderate confidence for association with resistance were considered false-susceptible isolates. Thus, due to the presence of these strains, 6 MDR-TB cases were not diagnosed by culture-based DST, 1 (1/19; 5.3% CI95: 0.1–26.0%) new case and 5 (5/64; 7.8% CI95: 2.6–17.3%) retreatment cases. The impact of the presence of these isolates on the outcome of RIF-based standard therapy could not be evaluated as all patients except one received second-line medications for TB treatment and the single patient that received RIF-based standard therapy died during treatment. Several authors have suggested lowering the RIF critical concentration to match the ECOFF of 0.25–0.5 μg/ml [19, 20]. However, some disputed isolates would still be missed, whereas some susceptible isolates could be misclassified as resistant. When possible, *rpoB* sequencing can help identify potentially underlying resistance-associated polymorphisms, especially among INH resistant strains [17].

Nonetheless, molecular methods designed to detect resistance have some limitations. First, none of the available molecular tests can detect all possible mutations or mechanisms (some are not yet identified) involved in resistance, and therefore, a variable percentage of resistant strain will not be detected. Previous studies have shown that 95–99% of mutations conferring resistance to RIF are associated with RRDR [2]. Mutations outside this region are rare, but not wholly exceptional. We found that 2.3% of RIF-resistant isolates did not contain any mutation in the *rpoB* gene, suggesting that other mechanisms of drug resistance must have been present, such as the action of efflux pumps. Due to the presence of these strains, 2 MDR-TB cases were not diagnosed by molecular techniques: 1 (1/19; 5.3% CI95: 0.1–26.0%) new case and 1 (1/64; 1.6% CI95: 0.0–8.4%) retreatment case. Another limitation stems from the fact that these methods cannot distinguish missense from silent mutations, and thus, a variable percentage of susceptible strains will be erroneously detected as resistant [21, 22]. None of the strains analyzed here had silent mutation.

Heteroresistance refers to the presence of drug-resistant and drug-sensitive microbial populations in the same sample, and it has been detected mainly using culture-based methods. The assumption of the agar proportion method is that TB drug resistance is denoted by ≥ 1% of colonies growing on drug-containing media as compared to drug-free medium, growing in a range of 1% up to 100% (completely resistant). Hence, heteroresistance is traditionally defined as 1–99% resistant colonies. This concept can be extrapolated to the field of molecular biology to describe wild-type and resistance-associated mutations occurring in the same sample [23]. This phenomenon has received little studied, but its prevalence is presumably dependent on the local resistance epidemiology. In the present study, 2.3% of heteroresistants strains were found among RIF-resistant isolates. Due to the presence of these strains, 2 MDR-TB cases (both retreatment cases; 2/64; 3.1% CI95: 0.4–10.8%) were not diagnosed by MTBDR*plus*. Simultaneous detection of wild-type and mutation bands with MTBDR*plus* suggest heteroresistance. However, in these cases heteroresistance was due to co-occurrence of wild-type and resistance-associated mutations not included in MTBDR*plus*; hence, it went unnoticed. These were incidental findings in our study and the prevalence of heteroresistance was likely underestimated. In other regions with a high TB burden the reported prevalence is 1.9–28.8% [23–25]. Other types of studies are needed to determine the prevalence of heteroresistance, and the analyses should be done on the primary samples or primary cultures [26].

In summary, 87 RIF-resistant strains were found, including 85.1% (74/87, CI95: 75.8–91.8%) with undisputed mutations, 10.3% (9/87, CI95: 4.8–18.7%) with disputed-mutations, 2.3% (2/87, CI95: 0.3–8.1%) with no *rpoB* mutations, and 2.3% (2/87, CI95: 0.3–8.1%) with heteroresistance. The following gene mutations were identified: 56.3% S450L, 17.2% D435V, 3.4% H445D, 3.4% H445L, 2.3% H445Y, 2.3% D435Y, 2.3% L452P, 2.3% L430P plus S493L, 1.1% Q432ins, 1.1% D435Y plus L452P, 1.1% N437del, 1.1% L449M plus S450F, 1.1% S450F, 1.1% S450W, 1.1% S450Y. This findings differ from those of previous reports, likely reflecting a dissimilar distribution of RIF resistance associated mutations in different geographical locations [27], or samples obtained in different phases of the MDR epidemic. Selective pressure induced by man-made mechanisms is the primary cause of MDR-TB. But, as the epidemic progresses and a high proportion of MDR TB cases occur by transmission, mutations that confer resistance without loss of reproductive fitness will likely be selected [28, 29]. It is important know the distribution of gene mutations associated with RIF resistance as it can affect the diagnostic performance of the techniques employed. In particular in this line, disputed RIF resistance mutations are clinically and epidemiologically relevant and occurs too frequently to be ignored. However, they are difficult to detect by the gold standard, culture-based methods. To increase their sensitivity, a lower RIF concentration could be used in these test or screening *rpoB* mutations in all MTB isolates is required. Nonetheless, for more accurate diagnosis of RIF resistance, not only detection of *rpoB* mutations, but also the characterization of the mutations detected is needed.

## Data Availability

The datasets generated and/or analysed during the current study are available in the NCBI SRA database repository, PRJNA727860.
